# Tubeworm May Live Longer by Cycling Its Sulfur Downward

**DOI:** 10.1371/journal.pbio.0030108

**Published:** 2005-02-22

Any organism may be limited by some essential nutrient in short supply—nitrogen for a plant on poor soil, for instance, or iron for phytoplankton in the open ocean. For the long-lived tubeworm Lamellibrachia luymesi, what it needs most and has least is sulfide. L. luymesi lives clustered around hydrocarbon-releasing ocean floor seeps in the Gulf of Mexico, and it—or rather, its menagerie of internal bacterial symbionts—uses the high-energy sulfide the way plants use sunlight, extracting energy and releasing the waste products, in this case sulfate.

With a lifespan of up to 250 years, L. luymesi is among the longest-lived of all animals, but how it obtains sufficient sulfide to keep going for this long has been a mystery. In this issue, Erik Cordes and colleagues propose a model in which, by releasing its waste sulfate not up into the ocean but down into the sediments, L. luymesi stimulates the growth of sulfide-producing microbes, and ensures its own long-term survival.[Fig pbio-0030108-g001]

**Figure pbio-0030108-g001:**
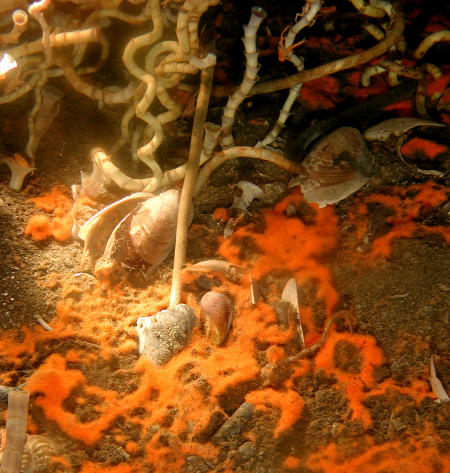
An aggregation of Lamellibrachia luymesi in the Gulf of Mexico (Photo: Ian MacDonald)

The sulfide L. luymesi needs is created by a consortium of bacteria and archaea that live in the sediments surrounding the vent. These chemoautotrophs use energy from hydrocarbons to reduce sulfate to sulfide, which L. luymesi absorbs through its unique “roots,” extensions of its body that it tunnels into the sediments. Measurements of sulfide and sulfate fluxes in the water near the vents are inconsistent with the observed tubeworm colony size and individual longevity, leading Cordes et al. to propose that L. luymesi also uses its roots to release sulfate back to the microbial consortia from which it draws its sulfide.

Without this return of sulfate, the model predicts an average lifespan of only 39 years in a colony of 1,000 individuals; with it, survival increases to over 250 years, matching the longevity of actual living tubeworms. The model, which was based largely on empirical data, is relatively unperturbed by changes in hydrocarbon seep rate, or in the growth and recruitment rates for the colony. The authors note that the proposed return of sulfate into deep sediments would, in theory, increase the local rate of carbonate rock formation, creating a barrier to fluid circulation into the sediments. Their model predicts this to occur after about 50 years, in line with observed reductions in tubeworm recruitment in colonies of this age. They propose that carbonate precipitation may be inhibited if roots can also release hydrogen ions, a possibility open to further testing. Their model also explains several biogeochemical anomalies observed near tubeworm colonies, including elevated levels of highly degraded hydrocarbons and higher than predicted rates of sulfur cycling.

To date, the proposed return of sulfate to the sediments through the roots is only a hypothesis—albeit one with much to support it—that still awaits direct confirmation. By providing a model in which this hypothetical interaction provides real benefits and explains real observations, the authors hope to stimulate further research into the biology of L. luymesi. For more on tubeworms, see “Microfauna–Macrofauna Interaction in the Seafloor: Lessons from the Tubeworm” (DOI: 10.1371/journal.pbio.0030102), also in this issue.

